# Tensor Decomposition for Spatial—Temporal Traffic Flow Prediction with Sparse Data

**DOI:** 10.3390/s20216046

**Published:** 2020-10-24

**Authors:** Funing Yang, Guoliang Liu, Liping Huang, Cheng Siong Chin

**Affiliations:** 1 School of Management, Jilin University, Changchun 130012, China; yfn@jlu.edu.cn (F.Y.); glliug@jlu.edu.cn (G.L.); 2 College of Computer Science and Technology, Jilin University, Changchun 130012, China; 3 School of Electrical and Electronic Engineering, Nanyang Technological University, Singapore 639798, Singapore; 4 Faculty of Science, Agriculture, and Engineering, Newcastle University Singapore, Singapore 599493, Singapore; cheng.chin@newcastle.ac.uk

**Keywords:** tensor decomposition, traffic flow, sparse data, traffic correlation pattern

## Abstract

Urban transport traffic surveillance is of great importance for public traffic control and personal travel path planning. Effective and efficient traffic flow prediction is helpful to optimize these real applications. The main challenge of traffic flow prediction is the data sparsity problem, meaning that traffic flow on some roads or of certain periods cannot be monitored. This paper presents a transport traffic prediction method that leverages the spatial and temporal correlation of transportation traffic to tackle this problem. We first propose to model the traffic flow using a fourth-order tensor, which incorporates the location, the time of day, the day of the week, and the week of the month. Based on the constructed traffic flow tensor, we either propose a model to estimate the correlation in each dimension of the tensor. Furthermore, we utilize the gradient descent strategy to design a traffic flow prediction algorithm that is capable of tackling the data sparsity problem from the spatial and temporal perspectives of the traffic pattern. To validate the proposed traffic prediction method, case studies using real-work datasets are constructed, and the results demonstrate that the prediction accuracy of our proposed method outperforms the baselines. The accuracy decreases the least with the percentage of missing data increasing, including the situation of data being missing on neighboring roads in one or continuous multi-days. This certifies that the proposed prediction method can be utilized for sparse data-based transportation traffic surveillance.

## 1. Introduction

With the prevalence of vehicles traveling on urban roads, the surveillance of transport traffic is an essential and important task for urban transportation management [1]. Obtaining accurate information about near-term future traffic flows of road segments in a traffic network has a wide range of applications, including vehicle navigation, congestion management [2,3,4], and understanding the urban structure [5]. A major problem in getting traffic flow information in real-time is that the vast majority of links are not equipped with traffic sensors, e.g., inductive loop detectors and closed-circuit cameras [6]. It is prohibitive to densely adopt them on the city scale, which consequently limits the coverage of traffic flow surveillance. Though trajectory data generated by probe vehicles for traffic surveillance does exist, there are still roads that are not traveled by GPS (global positioning system) equipped vehicles, on which the traffic cannot be monitored [7]. This directly makes the traffic flow prediction of a road network face the problem of data sparsity [8]. Based on the sparse sensing data, the problem of how to predict traffic flow effectively and efficiently has resulted in a tremendous amount of research. The majority of the previous investigations focus on short-term traffic prediction, using the full data on given road segments or specific areas without considering the data missing on continuous days. Another situation is the data missing on cascading road segments, which have not been well tackled.

Factually, the data sparsity from the spatial–temporal view makes the prevalent transportation traffic prediction method invalid, as it needs as original data (or extracted features) inputs and training process, such as the time series-based methods [9], the machine learning-related methods [10], and even the conventional neural network-based methods [11,12], or the prevalent deep learning methods [13,14].

There have been a significant number of studies investigating traffic dynamics with a focus on describing and understanding the resulting spatial–temporal traffic patterns [15,16]. Urban transportation traffic surveillance and prediction are the basis of transportation congestion identification [17] and travel path recommendation [18]. Researchers have made efforts to combine statistical methods [19] and machine learning methods [20,21]. Data-driven traffic surveillance methods are now mainly faced with the data sparsity problem [22,23], which cannot be solved by statistical models and machine learning methods.

Taking the traffic flow correlation between neighboring roads or directly connected roads into consideration, researchers turn to mining the spatial relationship between road segments [24] and utilizing the correlation findings to model urban transportation traffic [25,26,27]. This correlation analysis identifies the underlying relationship in the road networks, while a few studies have been conducted that make use of these findings to realize traffic prediction and solve the sparsity problems.

Among the effective methods to solve data sparsity are generative models, such as the tensor decomposition method [28]. Tensor decomposition is a generative method that can realize prediction with historical missing data [29]. Owing to the capability of tackling the sparsity problem, the tensor decomposition method has been adopted to model urban road network traffic prediction. Tang K. et al. [30] propose to construct a three-order tensor to model the travel time of different road segments under different traffic conditions in some time slots. This research takes the congestion status of road segments into the travel time modeling, while the temporal pattern is not given enough analysis. The research in [31] utilized the tensor decomposition method to recognize traffic patterns without predicting the future transportation traffic value, which is the main concern of transportation traffic modeling. 

Pastor G. et al. [32] capitalize on the definition of rank, called the tensor train, to present a low-rank tensor model for vehicular traffic volume data. The proposed method exploits all the correlations between local structures that are presented in the multiple models. The resulting optimally balanced tensor improves the imputation accuracy of the tensor train rank. However, this research focuses on the tensor rank and the imputation accuracy [33] and is not particularly concerned with traffic flow prediction. With tensor construction and tensor decomposition-based transportation traffic prediction, context-aware information about road network traffic can be captured and can assist in the improvement of traffic flow prediction accuracy [34,35,36,37]. This is the main focus of this paper, and we propose to model transportation traffic with a fourth-order tensor by incorporating the location correlation and the temporal dimension, including the time of day, the day of the week and the week of the month. 

By analyzing the historical traffic flow, we observe that the traffic flow presents a spatial pattern and temporal pattern. Specifically, the spatial pattern means that the traffic flow of cascading locations is of a similar variation trend or opposite trends. The temporal pattern is the periodicity and the temporal variation trend. The problem of how to incorporate these spatial and temporal correlations, which are hidden in the original data in traffic flow prediction models, has yet to be solved. Fully incorporating the underlying spatial and temporal correlation of transportation traffic data assists us in grasping the traffic pattern and alleviating the data sparsity problem during the traffic flow prediction process, which is considered in our proposed traffic flow prediction method.

Previous studies about transportation traffic correlation concentrate on the traffic correlation on different road segments [6]. These methods and findings assist us in understanding the road structure and transportation traffic surveillance. However, there is still not a sufficient transportation correlation for traffic flow prediction when faced with the data sparsity problem. The researchers in [15] proposed the utilization of a tensor-based method for modeling traffic data and completing the missing traffic data, whereas our motivation is to predict the traffic flow when the original data have different percentages of missing values. Additionally, the aforementioned study only focused on dealing with the missing traffic data at a single point. The case of missing data from neighboring roads was not considered, so it ignored the spatial correlation of traffic data.

In this paper, we propose to construct a traffic flow tensor that captures the spatial and temporal correlation of transportation traffic. We further propose a tensor decomposition method that predicts future traffic flow with missing data on cascaded road segments or on continuous days to tackle the spatial and temporal data sparsity problem. To be specific, the main contributions of this paper include the following aspects:

(1) We propose the utilization of a fourth-order tensor to model traffic flows, which can capture the spatial and temporal pattern of transportation traffic. Based on the tensor representation of traffic flows, we further propose a correlation estimation model to estimate the relationship between each pair of dimensions of the fourth-order tensor.
(2) Based on the constructed fourth-order traffic flow tensor, we propose a gradient descent-based tensor decomposition algorithm for traffic flow prediction, which does not need pre-training and can tackle the data sparsity problem from spatial and temporal perspectives.
(3) To validate the traffic flow prediction method, case studies on two real-world datasets are constructed. The evaluation results demonstrate that our proposed traffic flow prediction method outperforms other baselines with missing temporal data and missing spatial data.

According to the research problem above, the rest of this paper is organized as follows: the Methods are presented in Section 2. Specifically, the tensor construction, the tensor decomposition, and the prediction algorithm are, respectively, described. The Datasets and Metrics are described in Section 3. Our experimental Results using two real-world datasets are presented in order to evaluate our proposed traffic flow prediction method in Section 4. A Discussion of our conclusions and future works is conducted in Section 5.

## 2. Methods

### 2.1. Tensor Construction

By analyzing the traffic flow, we find that the traffic pattern correlates to the road segment location, time of day, day of the week, and the week of the month. In this paper, we construct a fourth-order tensor.

Specifically, we analyze the correlation between the four dimensions to certify the multi-pattern of traffic flow on urban roads, which is suitable for tensor modeling. It also helps to understand the feasibility of tensor-based traffic flow modeling.

Firstly, we incorporate a correlation calculation formula, as shown in Equation (1), to estimate the relationship between each dimension in the constructed tensor.

(1) $$S_{n}=\frac{\sum_{i\geq j\geq 1}^{col_{n}}R_{n}(i,j)}{col_{n}(col_{n}-1)/2}$$

where $R_{n}$ represents the correlation matrix after transforming the fourth-order traffic flow tensor into an n-order matrix, $col_{n}$ denotes the column number after transforming the traffic flow tensor into an n-order matrix, and $S_{n}$ represents the correlation results of the n-dimension of the traffic flow tensor. A larger value of $S_{n}$ represents a more correlated relation in the dimension. This correlation estimation model can certify that the traffic flow tensor is reasonably constructed and incorporates effective traffic pattern information underlined in the traffic flow data.

After transforming the fourth-order traffic flow tensor $X^{time\times day\times week\times location}$ into a matrix on the n-dimension, the matrix is constructed by columns on the n-dimension to get $X_{\left(n\right)}$. Then, the correlation matrix based on this transformed matrix denotes the correlation between these transformed matrices. Elements in the correlation matrix are the quantized relationship between columns. The average value after summing the correlation matrix entries obtains the average correlation.

### 2.2. Tensor Decomposition

After constructing the fourth-order traffic flow tensor, we utilize the Tucker-based tensor decomposition approach to predict future traffic flow values, which could capture the multi-dimensional inherent correlation of traffic flows. The tensor decomposition is shown in Equation (2).

(2) $$X\leftarrow \Phi \times_{1}U_{Time}\times_{2}U_{Day}\times_{3}U_{Week}\times_{4}U_{Location}$$

where $X\in {\mathbb{R}}^{288\times 7\times 7\times 10}$ in the evaluation and we set each time slot as 5 min. Then, the time slot number of a day is equal to 288. The day number of a week is 7, and we select 10 locations. Then, the core tensor is $\Phi \in {\mathbb{R}}^{3\times 3\times 3\times 3}$, which reflects the relation between the factor matrix of $U_{Time}\;$, $U_{Day}\;$, $\;U_{Week}$ and $U_{Location}$, which, respectively, denote the components of traffic patterns in terms of time, day, week, and location. 

To gradually represent the tensor-based prediction method, we first introduce the matrix filling-based method. Then, this can be extended to the fourth-order tensor decomposition method.

**Figure 1:**
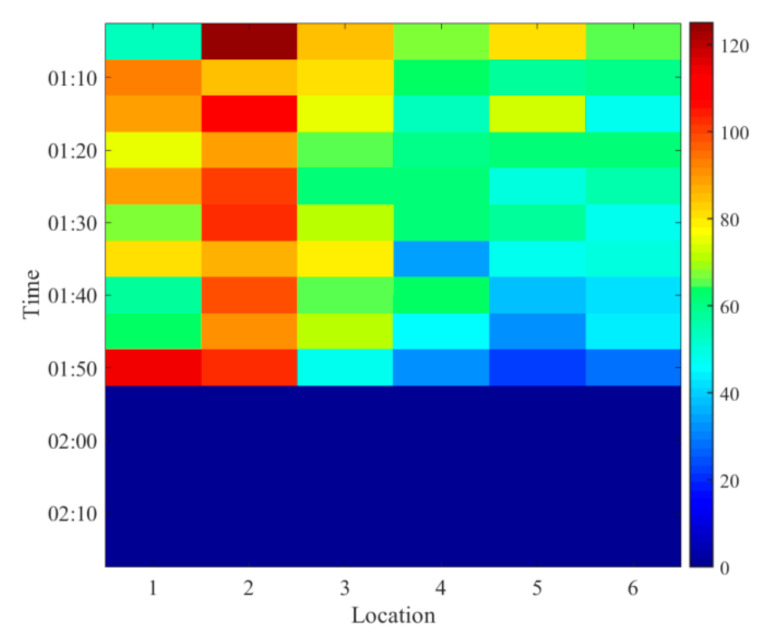
Traffic flow matrix.

We construct a two-order matrix that maps the spatial and temporal traffic flow, as shown in Figure 1. The dimension consists of location and time. Each entry of the matrix denotes the traffic flow value, and the empty entries mean values that are to be predicted. 

Generally, the matrix is represented as $X\in {\mathbb{R}}^{(\alpha \times h+\alpha \times p)\times l}$, where α means the total time length, and h,p, respectively, represent the historical time span and the prediction time span. l denotes the neighbor locations. For the prediction model, we assume that the current time slot is $t_{i}$, then the prediction problem is transformed to fill the values in the time span $\left[t_{i}+\alpha ,t_{i}+2\alpha ,\cdots ,t_{i}+p\times \alpha \right]$ given the known values in the time span $\left[t_{i},t_{i}-\alpha ,t_{i}-2\alpha ,\cdots ,t_{i}-h\times \alpha \right]$.

In fact, there has been sufficient research on filling missing values in matrices, such as the widely used SVD (singular value decomposition) algorithm. Matrix filling is generalized as an optimization problem, as shown in Equation (3).

(3) $$f_{W}(A,B)=\frac{1}{2}{\left\|X-AB^{T}\right\|}_{W}^{2}$$

where w is the weight matrix of the same size as matrix A. Each entry of w denotes whether the traffic flow exists. Specifically, 0 means a missing value (otherwise, values are 1). A and B are the factor matrices that need to be estimated using the factorizing algorithm. 

**Figure 2:**
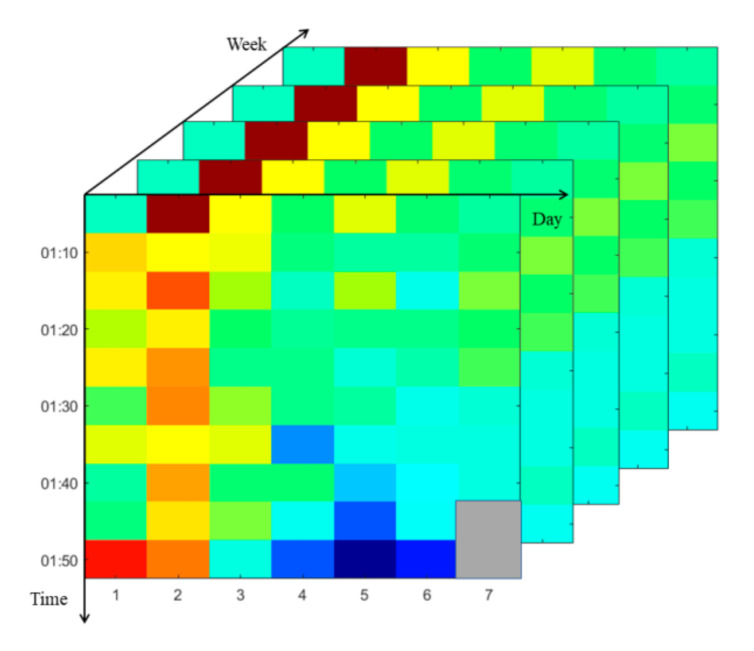
Third-order tensor.

Progressively, we illustrate a third-order tensor-based prediction method. The constructed tensor is shown in Figure 2. The three dimensions consist of the time, the day and the week.

**Figure 3:**
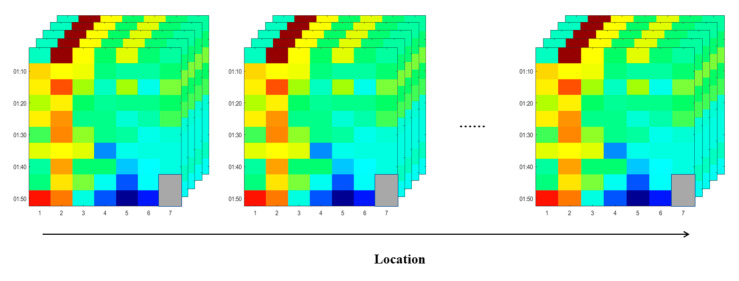
Fourth-order traffic flow tensor.

To incorporate the correlation between locations, we further add the location dimension and construct a fourth-order tensor of traffic flow, as shown in Figure 3. Finally, the constructed traffic flow tensor incorporates the spatial and temporal correlation.

### 2.3. Prediction Algorithm

In this part, a detailed representation of the tensor-based traffic flow prediction is given. As introduced, the tensor is factorized into the core tensor and factor matrices. The objective function is to minimize the original tensor and the multiplication of the core tensor and factor matrices. Note that we focus on sparsity-based traffic flow modeling. Thus, the tensor-based prediction algorithm could be capable of predicting future traffic flow values given several historical or continuous instances of missing data. The objective function used in this paper is shown in Equation (4).

(4) $$f_{W}(A,B,C)=\frac{1}{2}{\left\|W*X-W*⟦\Phi ;A,B,C⟧\right\|}_{W}^{2}$$

where W is the weight tensor of the constructed fourth-order traffic flow tensor X with the same size. Each element is defined as in Equation (5):

(5) $$w_{ijk}=\left\{ \begin{array}{ll} 0, & if\;x_{ijk}\;\mathrm{lost}\\ 1, & else; \end{array}\right.$$

According the objective function in Equation (4), the core tensor can be calculated as in Equation (6).

(6) $$\begin{array}{l} X=⟦\Phi ;U^{\left(1\right)},U^{\left(2\right)},\cdots ,U^{\left(N\right)}⟧\\ \Leftrightarrow \Phi =⟦X;U^{(1)T},U^{(2)T},\cdots ,U^{(N)T}⟧ \end{array}$$

To optimize the objective function in Equation (4) and get the factorized results of the core tensor and factor matrix *A*, *B*, *C*, we propose a weighted gradient descent-based optimization algorithm, the Tucker-WGopt (weighted gradient optimization) algorithm. For simplification, we first take a three-order tensor $X\in {\mathbb{R}}^{I\times J\times K}$ as an example. Then, the objective function described in Equation (4) can be transformed into the formula as shown in Equation (7).

(7) $$\begin{array}{l} f_{W}(A,B,C)=\sum_{i=1}^{I}\sum_{j=1}^{J}\sum_{k=1}^{K}w_{ijk}^{2}S\\ S=\left\{x_{ijk}^{2}-2x_{ijk}(\sum_{p=1}^{P}\sum_{q=1}^{Q}\sum_{r=1}^{R}\phi_{pqr}a_{ip}b_{jq}c_{kr})+{\left(\sum_{p=1}^{P}\sum_{q=1}^{Q}\sum_{r=1}^{R}\phi_{pqr}a_{ip}b_{jq}c_{kr}\right)}^{2}\right\} \end{array}$$

Then, the gradient of the factor tensor A can be calculated as shown in Equation (8).

(8) $$\begin{array}{l} \frac{\partial f}{\partial A}=\frac{\partial f}{\partial a_{ip}}=2\sum_{j=1}^{J}\sum_{k=1}^{K}w_{ijk}^{2}S\\ S=\{(-x_{ijk}+\sum_{l=1}^{P}\sum_{m=1}^{Q}\sum_{n=1}^{R}\phi_{imn}a_{il}b_{jm}c_{kr})(\sum_{q=1}^{Q}\sum_{r=1}^{R}\phi_{pqr}b_{jq}c_{kr})\} \end{array}$$

To simplify the representation in Equation (8), we introduce temporary variables, including a tensor Y, Y=W∗X, and Z=W∗⟦Φ;A,B,C⟧, then the objective function shown in Equation (4) can be transformed into Equation (9).

(9) $$f_{W}(A,B,C)=\frac{1}{2}{\left\|Y-Z\right\|}_{W}^{2}$$

Here, the temporary tensor Y can be pre-calculated to decrease the computation time and speed up the prediction algorithm. The gradient of each factor matrix can be represented as in Equation (10).

(10) $$\begin{array}{l} \frac{\partial f}{\partial A}=2(Z_{\left(1\right)}-Y_{\left(1\right)}){\left(\Phi \;\times_{2}B\;\times_{3}C\right)}_{\left(1\right)}^{T}\;;\\ \frac{\partial f}{\partial B}=2(Z_{\left(2\right)}-Y_{\left(2\right)}){\left(\Phi \;\times_{1}A\;\times_{3}C\right)}_{\left(2\right)}^{T}\;;\\ \frac{\partial f}{\partial C}=2(Z_{\left(3\right)}-Y_{\left(3\right)}){\left(\Phi \;\times_{1}A\;\times_{2}B\right)}_{\left(3\right)}^{T}\;; \end{array}$$

After extending it to the N-order tensor, the prediction algorithm is presented in Algorithm 1. The traffic flow value in the time span to be predicted is removed, and a certain percentage of the historical data before the prediction horizon are lost, meaning a sparse traffic flow tensor. The proposed traffic flow prediction algorithm can realize traffic flow forecasting by tackling this data sparsity problem.
**Algorithm 1.** Dynamic Traffic Flow PredictionInput: sparse traffic flow tensor **X** with data removed in the prediction horizon.Output: traffic prediction tensor $\tilde{X}$.1. Set the order of the core tensor Φ.2. Initialize the core tensor Φ.3. Initialize each factor matrix $A^{\left(1\right)},A^{\left(2\right)},\cdots ,A^{\left(N\right)};$4. Utilize the factor matrices to calculate the core tensor Φ, according to the formula in (6).5. Pre-calculate the tensor Y=W∗X with norm γ=‖Y‖.6. Calculate tensor $Z=W*⟦\Phi ;A^{\left(1\right)},\cdots ,A^{\left(N\right)}⟧$.7. Calculate the tensor T=Y-Z.8. Calculate $f=\frac{1}{2}\gamma -<Y,Z>+\frac{1}{2}{\left\|Z\right\|}^{2}$.9. Calculate the gradient matrix as $G^{\left(n\right)}=-T_{\left(n\right)}{\left(\Phi \times_{1}A^{\left(1\right)}\times \cdots \times_{n-1}A^{\left(n-1\right)}\times_{n+1}A^{\left(n+1\right)}\times \cdots \times_{N}A^{\left(N\right)}\right)}_{\left(n\right)}^{T}$10. Utilizing the gradient decent algorithm to get the core tensor Φ and the factor matrices $A^{\left(1\right)},\cdots ,A^{\left(N\right)}$.11. Calculate the filled tensor $\;\tilde{X}=\Phi \times_{1}A^{\left(1\right)}\times_{2}A^{\left(2\right)}\times \cdots \times_{N}A^{\left(N\right)}$12. Return $\tilde{X}$

## 3. Datasets and Metrics

In this section, we present the datasets in this study to verify the traffic prediction approach. Additionally, the metrics for measuring the prediction accuracy are elaborately described.

### 3.1. Datasets

In order to ensure the feasibility, repeatability, and usability of our model, we utilize two real-world datasets to test the performance of our traffic flow prediction algorithm. 

**Figure 4:**
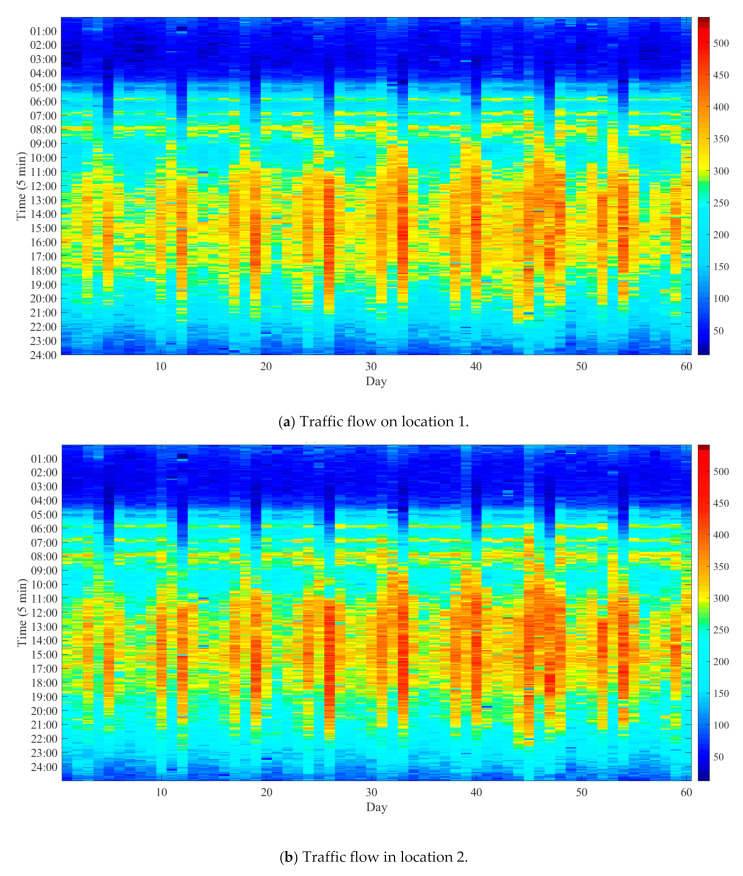
Traffic flow of two express ways.

Our dataset is the expressway traffic flow collected from the PeMS (performance measurement system) in California, which is a free way performance measurement system for all of California. It contains I-5 North roads, and the time spans from 1 March 2017 to 30 April 2017. Each time slot is 5 min, and the time span consists of 288 time slots. The dataset contains 11 locations. The first six-week dataset is used to train the model, and the last two-week dataset is applied to test the performance of prediction algorithms. For visualization, examples of two locations in the two months (60 days) are shown in Figure 4. 

This shows that the traffic flow of a location presents a variation pattern in the time of day and the day of the month, which can be captured using our proposed fourth-order tensor construction model.

**Figure 5:**
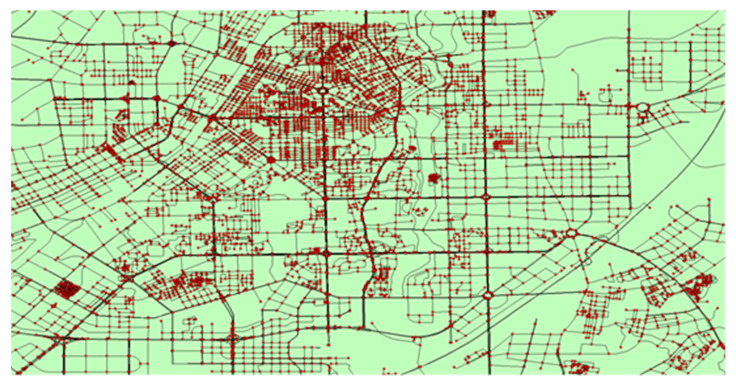
Case study area in Changchun, China, and the taxi trajectory distribution. The background is the road network, and the red points are the trajectory points (GPS points) generated by the taxi cabs. The trajectory is utilized to extract the traffic flow on a road.

Another dataset is the traffic flow on two cascading urban roads in Changchun, China. The traffic flow is obtained from urban taxi cabs traveling on roads. The GPS trajectories generated by the taxi cabs are utilized to extract the vehicle number on a road in a given time slot. The real dataset contains 1929 taxi cabs, and the trajectory data are generated from 1 May 2017 to 29 July 2017 with a total of 90 days of traffic flow data. The road network, together with the trajectory data of one day, are shown in Figure 5. In order to incorporate the spatial correlation of traffic flow between locations, we select two roads in the major area.

### 3.2. Performance Metrics

To quantify the prediction of traffic flow prediction algorithms, we apply four metrics, MAE (mean absolute error), MAPE (mean absolute percentage error), RMSE ((root mean square error)), and TCA (traffic capacity accuracy), which are shown from Equation (11) to Equation (14).

(11) $$MAE=\frac{1}{n}\sum_{t=1}^{n}\left|x_{t}-{\hat{x}}_{t}\right|$$

(12) $$MAPE=\frac{1}{n}\sum_{t=1}^{n}\frac{\left|x_{t}-{\hat{x}}_{t}\right|}{x_{t}}\times 100\%$$

(13) $$RMSE=\sqrt{\frac{1}{n}\sum_{t=1}^{n}{\left(x_{t}-{\hat{x}}_{t}\right)}^{2}}$$

(14) $$TCA=1-\frac{\left\|\left(I-W)*(X-\hat{X}\right)\right\|}{\left\|(I-W)*X\right\|}$$

where $x_{t}$ denotes the ground truth of traffic flow, ${\hat{x}}_{t}$ denotes the prediction value, and *n* is the sample number. $\hat{X}$ is the predicted tensor where the prediction horizon is along the timeline. Note that a smaller value of either MAE, MAPE, or RMSE indicates a better prediction. A bigger value of TCA instead represents a better prediction. Compared to the other three metrics, i.e., MAE, MAPE and RMSE, TCA incorporates the missing data information into measuring the prediction performance, which leads to its capability of verifying the prediction accuracy in terms of different levels of missing data in the model inputs. 

To demonstrate the advantage of our proposed traffic flow prediction algorithm, Tucker-WGopt, four baselines are adopted in the experiment. The baselines include Tucker-ALS (alternating least squares), CP (CANDECOMP/PARAFAC)-ALS, SVR (support vector regression), and the traditional time series model ARIMA (auto regressive integrated moving average).

## 4. Results and Analysis

In this section, we present the prediction performances for both the complete data and the sparse data in terms of prediction accuracy measured by the metrics. Moreover, we look into and compare time consumption for different methods in this section. Two real-world datasets are utilized in the experiment. The dataset for the Changchun, China, only contains two cascading roads, and the PeMS dataset contains 11 locations. In the experimental part, to evaluate the prediction performance on the complete data, we utilize both complete datasets. For evaluating the prediction accuracy in terms of sparse data, we utilize the PeMS dataset, as we should evaluate missing data in neighboring locations, and the PeMS dataset contains more neighboring locations. 

### 4.1. Correlation of Tensor Dimension Analysis

In this section, we present the correlation of the fourth-order tensor of traffic flow using our proposed correlation estimation model. The correlation calculation results are shown in Table 1. 

It can be found that the correlation in the location dimension is calculated to be the largest value. The temporal traffic pattern in the day of the week and the week of the month presents an adequate correlation, whereas the correlation in the time dimension has the smallest value. By looking into the real-world situation, this underlines that the traffic flow of a given day is a bit stochastic. When it comes to the long-term traffic pattern, say daily or weekly, the traffic instead presents a characteristically more stable pattern. Such stable patterns are captured in the tensor construction, and could facilitate the traffic prediction.

### 4.2. Prediction Performance with the Complete Data

We first test the prediction performance of the proposed model on the original datasets. Both original datasets are complete, meaning that no elements are missing. Such an evaluation helps us know how the model performs for a dataset where no elements are missing. Measured by three metrics, i.e., MAPE, RMSE, and TCA, the prediction results on the PeMS dataset and the trajectory dataset in Changchun, i.e., TraCC (trajectory dataset of Changchun, China), are shown in Table 2. 

The results in Table 2 show that the proposed algorithm, Tucker-WGopt, obtains the smallest prediction error measured by two metrics, namely MAPE and RMSE. The results of the two datasets are quite similar. In addition, it achieves the largest TCA value. This means that Tucker-WGopt outperforms other methods in terms of prediction accuracy. Compared to Tucker-ALS, the proposed Tucker-WGopt also obtains improvements, which verify the effectiveness of incorporating weighed gradient optimization into the Tucker-based tensor decomposition. Note that the TCA value obtained by the proposed method also performs the best when considering the missing data weight matrix. Further testing of the prediction performance with the sparse data is presented in the next part of this section. Aside from the prediction accuracy, we further show the performance from the perspective of computation time, which is shown in Table 3.

Table 3 shows that the proposed prediction algorithm, Tucker-WGopt, consumes less time compared to ARIMA. However, it does not show an advantage in terms of computing speed compared to Tucker-ALS, CP-ALS, or SVR. The advantage of the tensor decomposition or matrix decomposition models is that they do not require a training process. Moreover, the tensor-based methods are capable of tackling sparse data for value prediction. Sparse data mean that a certain number of entries in the tensor are missing, and the tensor decomposition method keeps being capable of predicting the future values even with these empty entries; meanwhile, such empty entries can be filled after utilizing the prediction algorithm. In the next section, we further verify the performance of traffic prediction when different levels of missing data exist.

### 4.3. Verifying with Sparse Data

As we need calculate the prediction accuracy of the model, we have collected datasets where the original data are not missing. The missing data are constructed by randomly removing data, which is carried out to test the model’s robustness in terms of missing data. Thus, the location of missing data exists randomly in the road network, and such stochastically generalized locations could help ensure the robustness of missing data for randomly selected roads. 

**Figure 6:**
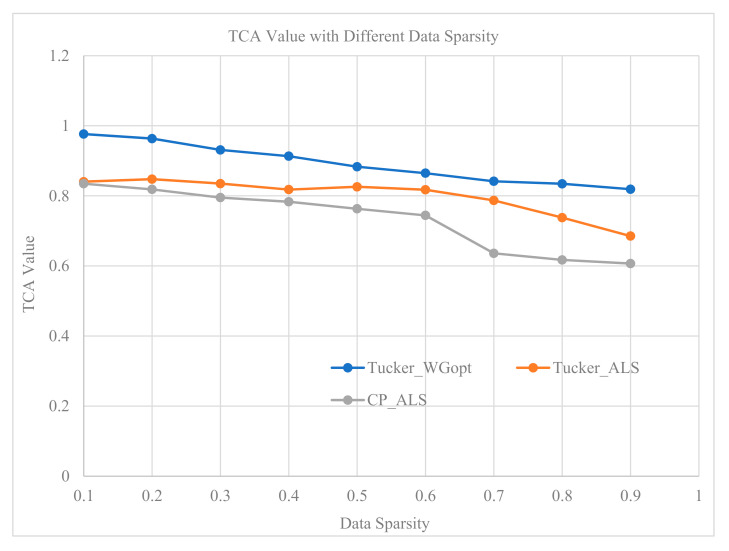
TCA (traffic capacity accuracy) with different percentages of missing data.

To further test the performance of our model with different amounts of missing data, we randomly remove values in the tensor. The percentage of removed data spans from 10% to 90%. Under such a situation, the prediction accuracy, measured by TCA, is as shown in Figure 6.

As shown in Figure 6, we found that the prediction accuracy decreases with the percentage of missing data increasing. The TCA values keep being larger than 0.8 even when 90 percent of data have been removed. This demonstrates that the proposed tensor decomposition-based method realizes an effective traffic flow prediction; moreover, it outperforms two other baselines under all percentages of missing data. 

**Figure 7:**
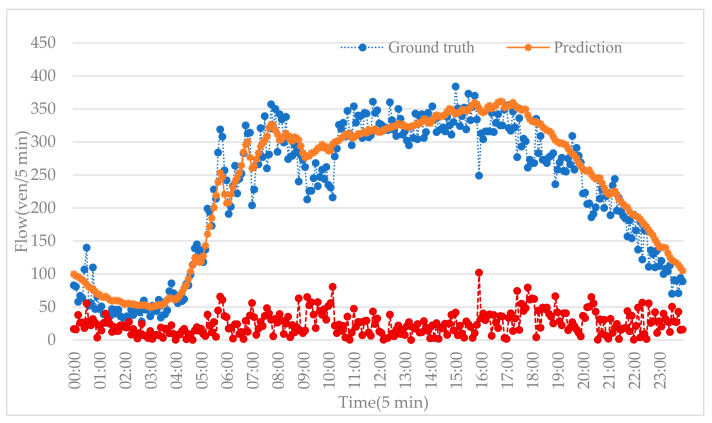
Prediction of our method measured by MAE (mean absolute error) with continuous missing data in one day.

In a real situation, missing data may exist in continuous time slots. For instance, where the data for a day are completely missing. To further verify the capability of tackling the missing data phenomenon for traffic prediction in the proposed method, we then delete the data in the day dimension, meaning that a whole day of data is missing. Measured by MAE, the results of continuous missing data in one day are shown in Figure 7. 

**Figure 8:**
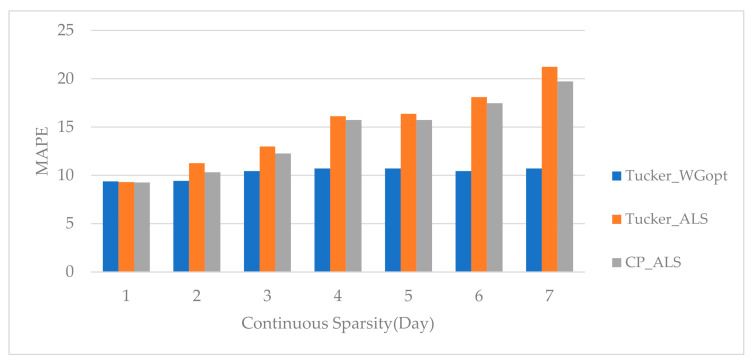
Prediction accuracy measured by MAPE (mean absolute percentage error) with continuous missing data in multi-days.

Measured by MAE, we find that the proposed method effectively predicts traffic flow values with small errors. For further testifying the robustness of the model in terms of tackling sparse data for prediction, we remove data for continuous days. Measured by MAPE, the results of three tensor-based methods are shown in Figure 8. We find that the results of the three methods are similar when the missing data are from less than three continuous days, whereas, with data removed for continuous days, the prediction errors of Tucker-ALS and CP-ALS increase substantially. However, the proposed prediction method, Tucker-WGopt, keeps a similar prediction accuracy, which is much smaller than the results of other two baselines. Such observations demonstrate that the proposed Tucker-WGopt algorithm facilitates the tensor decomposition to capture more spatiotemporal information on traffic flows for future traffic prediction. Hereby, the proposed method presents advantages over two other baselines for tackling data missing in continuous days.

**Figure 9:**
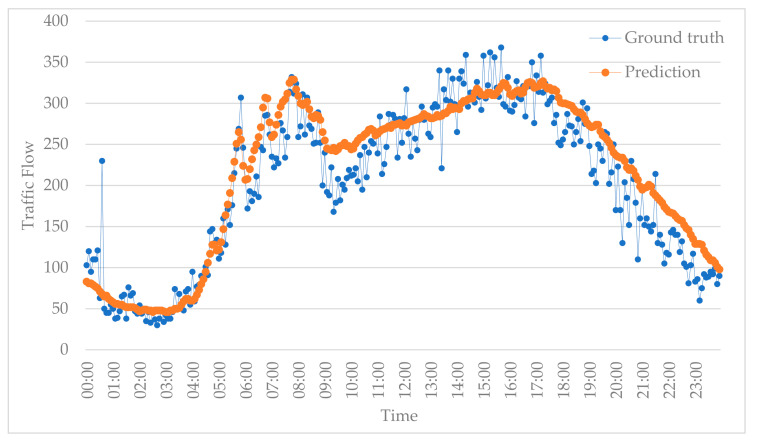
Prediction results with missing data on neighboring roads.

Another missing data scenario is that the data are missing on neighboring road segments. Here, we further test the prediction of our proposed method in this situation; the result is shown in Figure 9. This shows that the prediction algorithm effectively fits real traffic flow.

## 5. Discussion

This paper presents a tensor decomposition-based urban transportation traffic prediction method, which incorporates the location correlation of road segments in the road network and the temporal correlation into tensor construction. The temporal correlation contains the time of day, the day of the week, and the week of the month. In addition to the location of a road, a fourth-order tensor is constructed. Before tensor construction, we propose a traffic correlation estimation method to certify the rationality of the constructed tensor. After constructing the fourth-order traffic flow tensor, we further propose a tensor decomposition-based traffic flow prediction algorithm, which is capable of tackling missing data on neighboring road segments and on continuous days. Evaluating real-world datasets of traffic flow, it demonstrates that the proposed traffic flow method outperforms the existing baselines with different levels of data sparsity.

The proposed fourth-order tensor can be transformed into a fifth-order tensor only when we have multiple months of traffic data. By adding the dimension of the month, the temporal pattern in different months can be captured to predict traffic flows. Similarly, if we can obtain access to a dataset that covers more seasons or more years, the seasonal dimension or the yearly dimension can also be added. Such verification experiments could only be carried out when a much bigger dataset is available. Additionally, testing the approach on more road networks in other countries or cities may help further evaluate the effectiveness, a task which is left to our future studies.

In conclusion, this paper proposed to utilize tensor decomposition to model traffic flow and proposed a traffic flow prediction algorithm. The proposed method is designed mainly to tackle traffic flow prediction with missing data. In future studies, we will focus on fifth-order or sixth-order tensor modeling for traffic flow prediction. On the other hand, the context-aware information of traffic congestion status could also be incorporated into modeling the traffic flow at the road network level. 

## Figures and Tables

**Table 1:** Correlation between dimensions.

Dimension.	Matrix Size	Correlation
Time	288×(7×7×10)	0.41
Day	7×(288×7×10)	0.88
Week	7×(288×7×10)	0.96
location	10×(288×7×7)	0.98

**Table 2:** Effectiveness of different prediction methods.

Method	MAPE		RMSE		TCA	
	PeMS	TraCC	PeMS	TraCC	PeMS	TraCC
Tucker-WGopt	0.091392	0.091318	6.2138	6.2093	0.9866	0.9874
Tucker-ALS	0.097251	0.097039	8.8694	8.8439	0.9783	0.9832
CP-ALS	0.099368	0.099230	8.7244	8.7193	0.9774	0.9796
SVR	0.100135	0.100141	9.1256	9.1231	not applicable	
ARIMA	0.102438	0.102174	9.3695	9.3597	not applicable	

**Table 3:** Efficiency of different methods.

Method	Time (s)	Training Needed
Tucker-WGopt	5.1	N
Tucker-ALS	0.7391	N
CP-ALS	0.6773	N
SVR	0.3158	Y
ARIMA	13.7808	Y

## References

[1] Abadi A., Rajabioun T., Ioannou P.A. (2014). Traffic Flow Prediction for Road Transportation Networks with Limited Traffic Data. IEEE Trans. Intell. Transp. Syst..

[2] Çolak S., Lima A., González M.C. (2016). Understanding congested travel in urban areas. Nat. Commun..

[3] Olmos L.E., Çolak S., Shafiei S., Saberi M., González M.C. (2018). Macroscopic dynamics and the collapse of urban traffic. Proc. Natl. Acad. Sci. USA.

[4] Zeng G., Li D., Guo S., Gao L., Gao Z., Stanley H.E., Havlin S. (2018). Switch between critical percolation modes in city traffic dynamics. Proc. Natl. Acad. Sci. USA.

[5] Huang L., Yang Y., Gao H., Zhao X., Du Z. (2018). Comparing Community Detection Algorithms in Transport Networks via Points of Interest. IEEE Access.

[6] Liu Z., Li Z., Li M., Xing W., Lu D. (2016). Mining Road Network Correlation for Traffic Estimation via Compressive Sensing. IEEE Trans. Intell. Transp. Syst..

[7] Quddus M., Washington S. (2015). Shortest path and vehicle trajectory aided map-matching for low frequency GPS data. Transp. Res. Part C Emerg. Technol..

[8] Huang L., Yang Y., Zhao X., Ma C., Gao H. (2018). Sparse Data-Based Urban Road Travel Speed Prediction Using Probabilistic Principal Component Analysis. IEEE Access.

[9] Luo X., Niu L., Zhang S. (2018). An Algorithm for Traffic Prediction Based on Imporved SARMA and GA. KSCE J. Civ. Eng..

[10] Tang K., Chen S., Khattak A.J. (2018). A Spatial–Temporal Multitask Collaborative Learning Model for Multistep Traffic Flow Prediction. Transp. Res. Rec. J. Transp. Res. Board.

[11] Tang J., Liu F., Zou Y., Zhang W., Wang Y. (2017). An Improved Fuzzy Neural Network for Traffic Speed Prediction Considering Periodic Characteristic. IEEE Trans. Intell. Transp. Syst..

[12] Ma Z., Koutsopoulos H.N., Ferreira L., Mesbah M. (2017). Estimation of trip travel time distribution using a generalized Markov chain approach. Transp. Res. Part C Emerg. Technol..

[13] Wu Y., Tan H., Qin L., Ran B., Jiang Z. (2018). A hybrid deep learning based traffic flow prediction method and its understanding. Transp. Res. Part C Emerg. Technol..

[14] Zhao J., Gao Y., Bai Z., Lu S., Wang H. (2019). Traffic speed prediciton under non-recurrent congesion: Based on LSTM method and Beidou navigation satellite system data. IEEE Intell. Transpotatrion Syst. Mag..

[15] Tan H., Feng G., Feng J., Wang W., Zhang Y.-J., Li F. (2013). A tensor-based method for missing traffic data completion. Transp. Res. Part C Emerg. Technol..

[16] Zhong C., Manley E., Arisona S.M., Batty M., Schmitt G. (2015). Measuring variability of mobility patterns from multiday smart-card data. J. Comput. Sci..

[17] Li D., Fu B., Wang Y., Lu G., Berezin Y., Stanley H.E., Havlin S. (2014). Percolation transition in dynamical traffic network with evolving critical bottlenecks. Proc. Natl. Acad. Sci. USA.

[18] Wang Y., Zheng Y., Xue Y. Travel time estimation of a path using sparse trajectories. Proceedings of the 20th ACM SIGKDD International Conference on Knowledge Discovery and Data Mining-KDD ’14, Association for Computing Machinery (ACM).

[19] Li Z., Jiang S., Li L., Li Y. (2019). Building sparse models for traffic flow prediciton: An empirical comparison between statistical heuristics and geometric heuristics for Bayesian network approaches. Transp. B-Transp. Dyn..

[20] Nicholas G., Dokolov V. (2017). Deep learning for short-term traffic flow prediction. Transp. Res. Part C.

[21] Lin F., Xu Y., Yang Y., Ma H. (2019). A Spatial-Temporal Hybrid Model for Short-Term Traffic Prediction. Math. Probl. Eng..

[22] Lu L., Wang J., He Z., Chan C.-Y. (2018). Real-time estimation of freeway travel time with recurrent congestion based on sparse detector data. IET Intell. Transp. Syst..

[23] Mulla A.K., Joshi A., Chavan R., Chakraborty D., Manjunath D. (2018). A Microscopic Model for Lane-Less Traffic. IEEE Trans. Control. Netw. Syst..

[24] Peng S., Shen Y., Zhu Y., Chen Y. (2019). A Frequency-Aware Spatio-Temporal Network for Traffic Flow Prediction. DASFAA 2019: Database Systems for Advanced Applications.

[25] Yang Z., Bing Q., Lin C., Yang N., Mei D. (2014). Research on Short-Term Traffic Flow Prediction Method Based on Similarity Search of Time Series. Math. Probl. Eng..

[26] Wang Q., Wang J., Yuan Y. (2018). Locality constraint distance metric learning for traffic congesiton detection. Pattern Recognit..

[27] Min W., Wynter L. (2011). Real-time road traffic prediction with spatio-temporal correlations. Transp. Res. Part C Emerg. Technol..

[28] Lin L., Li J., Chen F., Ye J., Huai J. (2017). Road traffic Speed Preediction: A Probabilistic Model Fusing Multi-Source Data. IEEE Trans. Knowl. Data Eng..

[29] Shao W., Chen L. (2018). License Plate Recognition Data-Based Traffic Volume estimation Using Collaborative Tensor Decomposition. IEEE Trans. Intellignet Transp. Syst..

[30] Tang K., Chen S., Liu Z. (2018). Citywide Spatial-Temporal Travel Time Estimation Using Big and Sparse Trajectories. IEEE Trans. Intell. Transp. Syst..

[31] Zhong H., Qi G., Guan W., Hua X. (2019). Application of Nonnegative Tensor Factorization for Intercity Rail–Air Transport Supply Configuration Pattern Recognition. Sustainability.

[32] Pastor G., Giancarlo P. (2018). A Low-Rank Tensor Model for Imputation of Missing Vehicular Traffic Volume. IEEE Trans. Veh. Technol..

[33] Chen X., He Z., Sun L. (2019). A Bayesian tensor decomposition approach for spatiotemporal traffic data imputation. Transp. Res. Part C Emerg. Technol..

[34] Tang K., Chen S., Khattak A. (2018). Personalized travel time estimation for urban road networks: A tensor-based context-aware approach. Expert Syst. Appl..

[35] Arena F., Ticali D. The development of autonomous driving vehicles in tomorrow’s smart cities mobility. Proceedings of the International Conference of Computational Methods in Sciences and Engineering 2018.

[36] Arena F., Pau G., Severino A. (2020). An Overview on the Current Status and Future Perspectives of Smart Cars. Infrastructures.

[37] Zhao J., Gao Y., Tang J. (2018). Highway Travel Time Prediction Using Sparse Tensor Completion Tactics and-Nearest Neighbor Pattern Matching Method. J. Adv. Transp..

